# Advanced Exergy-Based Analysis of an Organic Rankine Cycle (ORC) for Waste Heat Recovery

**DOI:** 10.3390/e25101475

**Published:** 2023-10-23

**Authors:** Zineb Fergani, Tatiana Morosuk

**Affiliations:** 1 Laboratory of Biomaterials and Transport Phenomena, Department of Process and Environmental Engineering, University of Medea, Medea 26000, Algeria; ferganizineb@gmail.com; 2 Institute for Energy Engineering, Technische Universität Berlin, Marchstr. 18, 10587 Berlin, Germany

**Keywords:** advanced exergy analysis, advanced exergoeconomics analysis, ORC, optimization

## Abstract

In this study, advanced exergy and exergoeconomic analysis are applied to an Organic Rankine Cycle (ORC) for waste heat recovery to identify the potential for thermodynamic and economic improvement of the system (splitting the decision variables into avoidable/unavoidable parts) and the interdependencies between the components (endogenous and exogenous parts). For the first time, the advanced analysis has been applied under different conditions: constant heat rate supplied to the ORC or constant power generated by the ORC. The system simulation was performed in Matlab. The results show that the interactions among components of the ORC system are not strong; therefore, the approach of component-by-component optimization can be applied. The evaporator and condenser are important components to be improved from both thermodynamic and cost perspectives. The advanced exergoeconomic (graphical) optimization of these components indicates that the minimum temperature difference in the evaporator should be increased while the minimum temperature difference in the condenser should be decreased. The optimization results show that the exergetic efficiency of the ORC system can be improved from 27.1% to 27.7%, while the cost of generated electricity decreased from 18.14 USD/GJ to 18.09 USD/GJ.

## 1. Introduction

Industrial sectors play an essential role in the economy of all countries. However, industrial sectors contribute enormously to environmental problems. Different technologies have been developed to reduce air pollution and greenhouse gas rates, for example, cleaner production processes, energy-efficient machinery, and waste reduction strategies. Waste heat recovery is a good and promising option for these technologies. The cement industry is an example [1,2,3,4,5] because of its energy-intensive processes and associated direct emissions.

Organic Rankine Cycle (ORC) is one of the most efficient waste heat recovery systems. It has known an increasing interest in recent years as it can achieve significant gains in energy, cost, and environment. Several studies have been conducted to investigate the performance of ORC systems using exergy-based analysis, which is one of the modern tools for evaluating energy conversion systems. Karellas et al. [6] compared the energetic and exergetic performance of the water steam cycle and ORC for waste heat recovery in a cement plant. The exergy analysis results indicated that the major exergy destructions were associated with the heat exchangers for both waste heat recovery (WHR) systems. Wang et al. [7] conducted a comparative study of an ORC and other cogeneration systems in the cement industry based on the exergy analysis. The results showed significant exergy destruction in the turbine, condenser, and heat recovery vapor generator. Energy and exergy analysis of an ORC for waste heat recovery in a cement factory was also investigated by Ustaoglu et al. [8]; the exergy destruction in the heat exchanger and evaporator presents 80% of the total exergy destruction. Fergani et al. [9,10] performed exergy, exergoeconomic, and exergoenvironmental optimization of the ORC with different working fluids, including the mixtures. The turbine and heat exchangers are the components that require attention regardless of the used working fluid.

Conventional exergy analysis can define the exergy destruction and exergetic efficiency of system components, and based on this information, the improvement potential is defined. However, conventional exergy analysis cannot reveal either interaction among the system components of the plant or estimate the real potential for system improvement (Morosuk and Tsatsaronis [11]).

An advanced exergy-based analysis (Morosuk and Tsatsaronis [12]) has been developed to overcome these limitations. In this type of analysis, the exergy destruction within each component can be divided into avoidable and unavoidable parts to provide information for improving the potential of each component. The exergy destruction can also be split into endogenous and exogenous parts; this splitting provides information on the interaction between system components. Recently, advanced exergy analyses have been successfully applied to various energy conversion systems. ORC is among these systems. Nami et al. [13] carried out conventional and advanced exergy analysis for a dual fluid ORC. They reported that the low-pressure turbine, low-pressure, and high-pressure vapor generator are the most critical. Galindo et al. [14] performed the advanced exergy analysis for an ORC coupled to an internal combustion engine. This analysis has shown that the cycle has great potential for improvement. Using the advanced exergoeconomic approach, Dai et al. [15] evaluated the energetic and economic performance of ORC using different hydrocarbon-based working fluids; they reported that the avoidable endogenous cost is relatively sensitive to the temperature of the heat source.

Based on the brief review, we can conclude that advanced exergy-based analyses are promising evaluation and optimization tools. There are few publications on applying advanced exergy-based analyses to ORC systems. Moreover, none of these publications have reported implementing the results obtained for optimization. In addition, no publications (to the authors’ best knowledge) where advanced exergy-based graphical optimization has been performed. In this paper, advanced exergy and exergoeconomic analyses were applied to evaluate the performance of an ORC system for waste heat recovery in a cement plant. The graphical optimization procedure was applied to the critical components to obtain conclusions important for the overall ORC system.

## 2. System Description and Modeling

**Figure 1:**
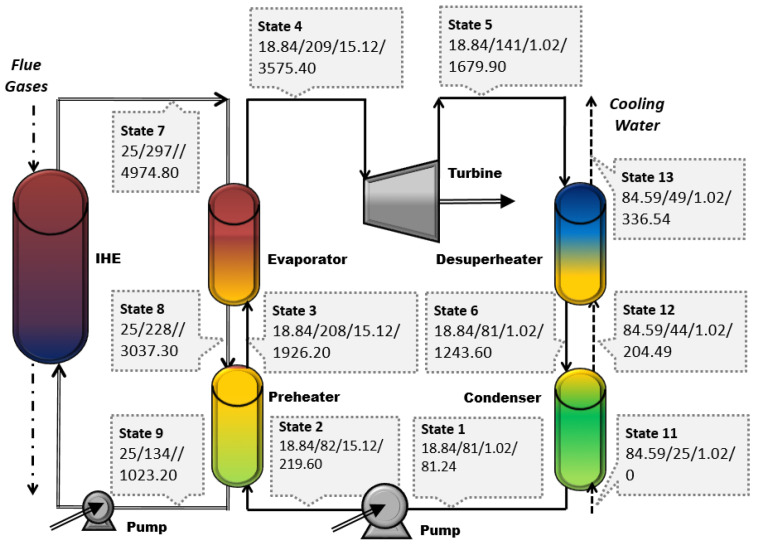
Schematic of the ORC system and the thermodynamic data for the Base Case operation conditions ($\dot{m}$ (kg/s)/T (°C)/p (bar)/$\dot{E\;}$(kW)).

Figure 1 shows a schematic of the waste heat recovery ORC system that consists of two sub-systems:

- Thermal oil circuit (states 7-8-9) to recover the waste heat from the clinker cooler exhaust airflow;
- The ORC system (states 1-…-6). Cyclohexane is the working fluid [9]. The ORC system consists of the evaporator block (preheater and evaporator), the turbine, the condenser block (condenser and desuperheater), and the pump. The liquid cyclohexane is the working fluid of the ORC. Detailed information about the selection of the working fluids, including the performance analysis, can be found, for example, in [16,17].

The liquid cyclohexane is heated and evaporated to 207 °C at a pressure of 15.1 bar and further expanded within the turbine. The expanded cyclohexane leaves the turbine at a temperature of 140 °C and a pressure of 1.02 bar; it desuperheated and condensed. Finally, the condensed working fluid returns to the evaporator via the pressurizing pump.

The evaluated system has been modeled using Matlab software (Matlab R2018b) (using Refprop software, Refprop 9.0, for thermodynamic properties of the working fluids) under the following assumptions: the system operates under steady-state conditions; the pressure drops and heat losses in heat exchangers and pipelines are neglected.

The energy balances for the ORC system components are as follows:

- Pump
  (1) $${\dot{W}}_{p}={\dot{m}}_{wf}(h_{2}-h_{1})$$
- Turbine
  (2) $${\dot{W}}_{t}={\dot{m}}_{wf}(h_{4}-h_{5})$$
- Evaporator
  (3) $${\dot{Q}}_{evp}={\dot{m}}_{wf}\left(h_{4}-h_{3}\right)={\dot{m}}_{HTO}{cp}_{HTO}\left(T_{7}-T_{8}\right)$$
- Preheater
  (4) $${\dot{Q}}_{pre}={\dot{m}}_{wf}\left(h_{3}-h_{2}\right)={\dot{m}}_{HTO}{cp}_{HTO}\left(T_{8}-T_{9}\right)$$
- Condenser
  (5) $${\dot{Q}}_{con}={\dot{m}}_{wf}\left(h_{6}-h_{1}\right)={\dot{m}}_{water}{cp}_{water}\left(T_{12}-T_{11}\right)$$
- Desuperheater
  (6) $${\dot{Q}}_{Desp}={\dot{m}}_{wf}\left(h_{6}-h_{5}\right)={\dot{m}}_{water}{cp}_{water}\left(T_{13}-T_{12}\right)$$

It should be noted that Fergani et al. [9] have previously validated the developed model (i.e., properties of the used working fluids); therefore, this part of the research is not reported here.

## 3. Conventional Exergy-Based Analysis

### 3.1. Exergy Analysis

The purpose of the conventional exergy analysis is to assess the performance of the system and identify the thermodynamic inefficiencies on a system and component level [18]. The exergy balance for the *k*th component can be written in terms of “exergy of fuel/exergy of product” as [19]:

(7) $${\dot{E}}_{F,\;k}={\dot{E}}_{P,\;k}+{\dot{E}}_{D,\;k}$$

${\dot{E}}_{F}$, ${\dot{E}}_{P}$, and ${\dot{E}}_{D}$ represent the exergy rate of the fuel, the product, and the destruction, respectively. The exergetic efficiency is calculated as

(8) $$\varepsilon_{k}=\frac{{\dot{E}}_{P,\;k}}{{\dot{E}}_{F,\;k}}$$

### 3.2. Exergoeconomic Analysis

Exergoeconomic analysis is the combination of economic and exergetic analysis; it aims to evaluate the cost-effectiveness of the system [19].

For the exergoeconomic analysis, cost balance equations with the auxiliary equations (if necessary) are applied for the kth component of the ORC cycle using the exergy costing principle $\dot{C}=c\dot{E}$ [18,19]

(9a) $$\sum {\dot{C}}_{out,k}=\sum {\dot{C}}_{in,k}+{\dot{Z}}_{\;k}$$

or in terms of “exergy of fuel/exergy of product”

(9b) $${\dot{C}}_{P,\;k}={\dot{C}}_{F,\;k}+{\dot{Z}}_{k}$$

The value of ${\dot{Z}}_{\;k}$ represents the total capital investment (*TCI*) and operating maintenance calculated using the Total Revenue Requirement method of the economic analysis [19]. The cost equations used to calculate *TCI_k_* are given in Table 1.

Exergoeconomic analysis provides several parameters that are important in developing the strategies for improving/optimizing the economic performance of the systems: the cost associated with the exergy destruction (${\dot{C}}_{D,k}=c_{F,k}{\dot{E}}_{D,k}$) and the total cost associated with the component (${\dot{Z}}_{\;k}+{\dot{C}}_{D,k}$).

For the conclusions about the optimization strategy, an exergoeconomic factor is recommended to be used [18,19]

(10) $$f_{k}^{}=\frac{{\dot{Z}}_{k}^{}}{{\dot{Z}}_{k}^{}+{\dot{C}}_{D,k}^{}}=\frac{{\dot{Z}}_{k}^{}}{{\dot{Z}}_{k}^{}+c_{F,k}{\dot{E}}_{D,k}^{}}$$

Up to now, the values of *f* reported in the literature for each component type are not systemized.

## 4. Advanced Exergy-Based Analysis

### 4.1. Advanced Exergy Analysis

To identify the real potential for improvement in the thermodynamic efficiency of a component, the total exergy destruction within the *k*th component should be split into unavoidable and avoidable parts [22]

(11) $${\dot{E}}_{D,\;k}={\dot{E}}_{D,k}^{UN}+{\dot{E}}_{D,k}^{AV}$$

The unavoidable exergy destruction (${\dot{E}}_{D,k}^{UN}$) is a part of the exergy destruction within the *k*th component that cannot be reduced due to technological limitations. In contrast, avoidable exergy destruction (${\dot{E}}_{D,k}^{AV}$) is a subject for the improvement procedure. If ${\dot{E}}_{D,k}^{UN}<{\dot{E}}_{D,k}^{AV}$, then the *k*th component should be considered for improvement. The absolute values play a role, particularly on the system level.

The unavoidable exergy destruction should be calculated assuming that the *k*th component operates under the highest possible performance (“best case”).

Splitting the exergy destruction into endogenous and exogenous parts provides information on the interactions among system components [11,12]

(12) $${\dot{E}}_{D,\;k}={\dot{E}}_{D,k}^{EN}+{\dot{E}}_{D,k}^{EX}$$

The endogenous exergy destruction (${\dot{E}}_{D,k}^{EN}$) represents the irreversibility within the *k*th component that operates under its real conditions, while all other components operate in an “ideal” way. The exogenous part is the exergy destruction (${\dot{E}}_{D,k}^{EX}$) in the *k*th component caused by remaining components.

The absolute values are secondary important to the relations:

- ${\dot{E}}_{D,k}^{EN}>{\dot{E}}_{D,k}^{EX}$—the interconnection between the components is not strong, and the approach of component-by-component optimization can be applied;
- ${\dot{E}}_{D,k}^{EN}<{\dot{E}}_{D,k}^{EX}$—the interconnection between the components is strong; therefore, the system optimization is meaningful;
- ${\dot{E}}_{D,k}^{EN}\approx {\dot{E}}_{D,k}^{EX}$—the interconnection between the components requires a deeper evaluation, i.e., further splitting the value of ${\dot{E}}_{D,k}^{EX}$ [11,12].

Several methods have been developed to calculate the value of ${\dot{E}}_{D,k}^{EN}$ [12].

The values of ${\dot{E}}_{D,k}^{UN,EN}$, ${\dot{E}}_{D,k}^{UN,EX}$, ${\dot{E}}_{D,k}^{AV,EN}$, and ${\dot{E}}_{D,k}^{AV,EX}$ denote the unavoidable endogenous exergy destruction, unavoidable exogenous exergy destruction, avoidable endogenous exergy destruction, and avoidable exogenous exergy destruction, respectively. Where ${\dot{E}}_{D,k}^{UN,EN}$ can be calculated as [11,12]

(13) $${\dot{E}}_{D,k}^{UN,EN}={\dot{E}}_{P,k}^{EN}{\left(\frac{{\dot{E}}_{D,k}}{{\dot{E}}_{P,k}}\right)}^{UN}$$

### 4.2. Advanced Exergoeconomic Analysis

For the advanced exergoeconomic analysis, the investment cost within the *k*th component is also split into unavoidable and avoidable parts [22]

(14) $${\dot{Z}}_{k}={\dot{Z}}_{k}^{UN}+{\dot{Z}}_{k}^{AV}$$

The unavoidable investment cost is found when *k*th component is operating under the lowest possible performance conditions (“worse case”). The value of the unavoidable cost rate can be obtained as follows:

(15) $${\dot{Z}}_{k}^{UN}={\dot{E}}_{P,\;k}{\left(\frac{{\dot{Z}}_{k}}{{\dot{E}}_{P,\;k}}\right)}^{UN}$$

Similarly, the cost rate associated with the exergy destruction is split into unavoidable and avoidable parts:

(16a) $${\dot{C}}_{D,\;k}={\dot{C}}_{D,\;k}^{UN}+{\dot{C}}_{D,\;k}^{AV}=c_{F,\;k}\left({\dot{E}}_{D,\;k}^{UN}+{\dot{E}}_{D,\;k}^{AV}\right)$$

thus,

(16b) $${\dot{C}}_{D,\;k}^{UN}=c_{F,\;k}{\dot{E}}_{D,\;k}^{UN}$$

The investment cost and the cost rate of the exergy destruction can also be split into endogenous and exogenous parts to show the cost interdependencies among the system components [11].

All the same considerations about combination and extensions in an advanced exergy analysis can be applied to an advanced exergoeconomic analysis; the investment cost and cost rate associated with the exergy destruction can be split into unavoidable endogenous, unavoidable exogenous, avoidable endogenous, and avoidable exogenous parts:

(17) $${\dot{Z}}_{k}={\dot{Z}}_{k}^{UN,EN}+{\dot{Z}}_{k}^{UN,EX}+{\dot{Z}}_{k}^{AV,EN}+{\dot{Z}}_{k}^{AV,EX}$$

(18) $${\dot{C}}_{D,k}=c_{F,\;k}\left({\dot{E}}_{D,\;k}^{UN,EN}+{\dot{E}}_{D,\;k}^{UN,EX}+{\dot{E}}_{D,\;k}^{AV,EN}+{\dot{E}}_{D,\;k}^{AV,EX}\right)$$

**Figure 2:**
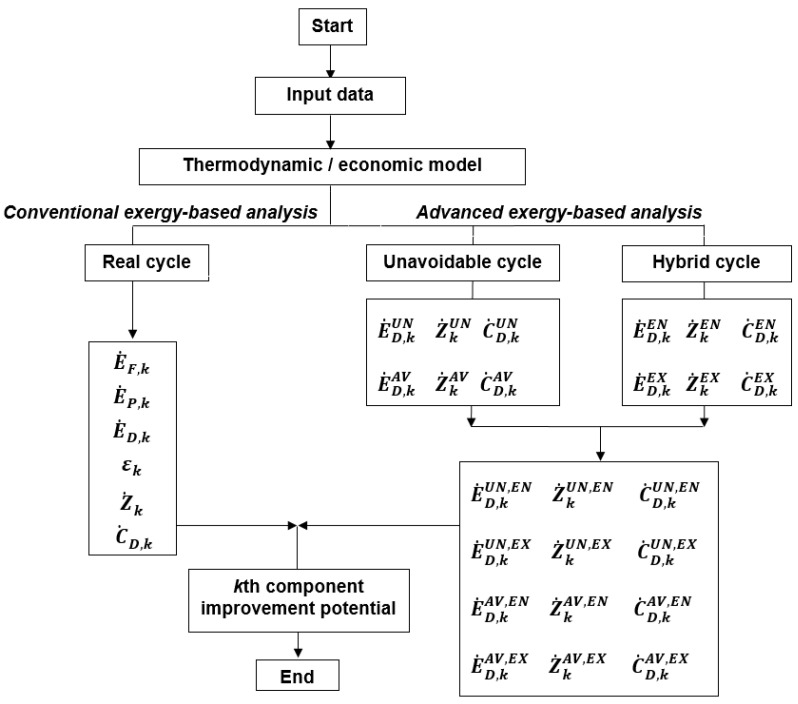
The calculation procedure.

The calculation procedure applied to the conventional and advanced exergy analysis is shown in Figure 2. Here, the so-called “thermodynamic cycle method” is applied where the endogenous values are calculated using a “hybrid cycle” and the unavoidable values using the “unavoidable cycles”. A very detailed application of the thermodynamic cycle method to the advanced exergetic analysis of the PCR can be found in [23].

The following variables: ${\dot{Z}}_{k}^{AV,EN}$, $C_{D,k}^{AV,EN}$, and $f_{k}^{AV,EN}$ can be used to provide a piece of comprehensive information from an advanced exergoeconomic analysis

(19) $$f_{k}^{AV,EN}=\frac{{\dot{Z}}_{k}^{AV,EN}}{{\dot{Z}}_{k}^{AV,EN}+{\dot{C}}_{D,k}^{AV,EN}}=\frac{{\dot{Z}}_{k}^{AV,EN}}{{\dot{Z}}_{k}^{AV,EN}+c_{F,k}{\dot{E}}_{D,k}^{AV,EN}}$$

## 5. Advanced Exergy-Based Optimization

**Figure 3:**
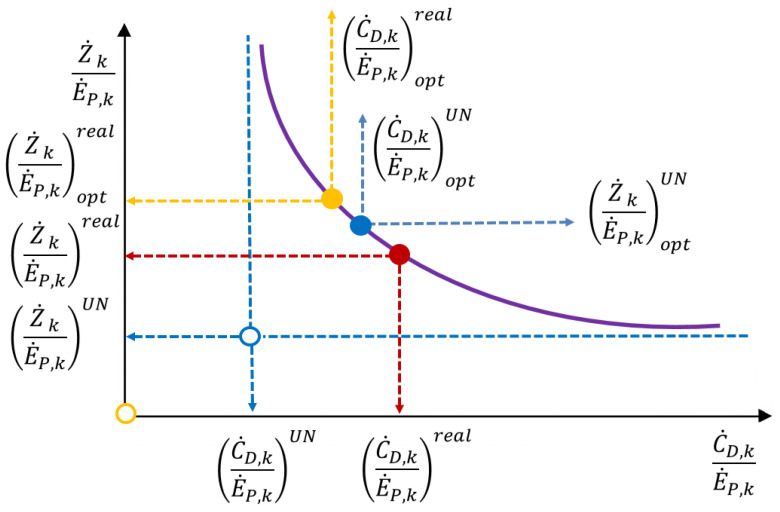
Graphic presentation of the advanced exergoeconomic optimization (adapted from [11]): in red—Base Case; in yellow—optimization based on conventional exergy-based analysis (exergoeconomic analysis, and in blue—optimization based on advanced exergy-based analysis.

Figure 3 illustrates the methodology for optimization based on advanced exergy and exergoeconomic analyses [11]. This is a trade-off model between thermodynamics and economics but in terms of the exergy-based methods. The *x*-axis represents the cost of exergy destruction per unit of product exergy. The *y*-axis represents the investment costs per unit of product exergy. The curve for each component is supposed to be developed with the help of the *TCI_k_* equations (Table 1). The “red dot” corresponds to the Base Case. Without applying the advanced exergy-based methods (*x* = 0 and *y* = 0), the optimal value corresponds to the “yellow dot”.

For advanced exergy-based optimization, the values ${\left(\frac{{\dot{C}}_{D,\;k}}{{\dot{E}}_{P,\;k}}\right)}^{UN}$ and ${\left(\frac{{\dot{Z}}_{\;k}}{{\dot{E}}_{P,\;k}}\right)}^{UN}$ should be excluded from the optimization procedure. Therefore, the adjusted *x*-axis and *y*-axis are applied and, therefore, adjusted optimal conditions as the “blue dot”.

Note that the location of “red dot”, “yellow dot”, and “blue dot” can be different depending on the system being evaluated, the type of component, and assumptions for calculating the ${\left(\frac{{\dot{C}}_{D,\;k}}{{\dot{E}}_{P,\;k}}\right)}^{UN}$ and ${\left(\frac{{\dot{Z}}_{\;k}}{{\dot{E}}_{P,\;k}}\right)}^{UN}$ value, etc.

## 6. Results and Discussion

### 6.1. Advanced Exergy Analysis

Conventional exergy and exergoeconomic analyses have already been reported by Fergani et al. [9]. The thermodynamic data for the Base Case are given in Figure 1. The main results of the conventional analysis are summarized in Table 2.

For the application of the advanced analysis, the main assumptions for real, unavoidable, and ideal conditions are listed in Table 3.

In this paper, for the first time, the advanced exergy and exergoeconomic analyses are conducted under different conditions:

- Power generated by ORC is constant (${\dot{W}}_{net}=const$);
- Heat supplied to the ORC is constant (${\dot{Q}}_{sys}=const$).

The results of the advanced exergy analysis are presented in Table 4. For splitting the variables into unavoidable/avoidable parts, the main assumptions (${\dot{W}}_{net}=const$ or ${\dot{Q}}_{sys}=const$) do not play a role because the simulation is conducted for each component in isolation from the entire system.

It can be seen that the only component with a higher unavoidable exergy destruction part (54%) is the evaporator. The avoidable exergy destruction rates in the turbine, condenser, and pump are higher than the unavoidable rate; this means that there are technical possibilities to improve these components. A total of 77% of the exergy destruction in the condenser could be avoided when the component operates with ${∆T}_{min}=5\;K$. Moreover, 70% and 75% of the exergy destruction in the turbine and pump could be avoided if the components operate with higher isentropic efficiencies of 90%.

It should be noted that the splitting of exergy destruction into endogenous/exogenous parts has different results depending on the conditions: ${\dot{Q}}_{sys}=const$ or ${\dot{W}}_{net}=const$. The differences are caused by changes in the mass flow rate of the working system under different initial conditions (a detailed explanation can be found in [11]). The splitting of exergy destruction into endogenous/exogenous parts shows that for all components, the value of endogenous exergy destruction is higher than the exogenous exergy destruction. This means that the interactions among the components are not strong. Therefore, to improve the system performance, the designer can follow the component-by-component approach. For ${\dot{Q}}_{sys}=const$, the endogenous exergy destruction values are a little higher than for ${\dot{W}}_{net}=const$.

**Figure 4:**
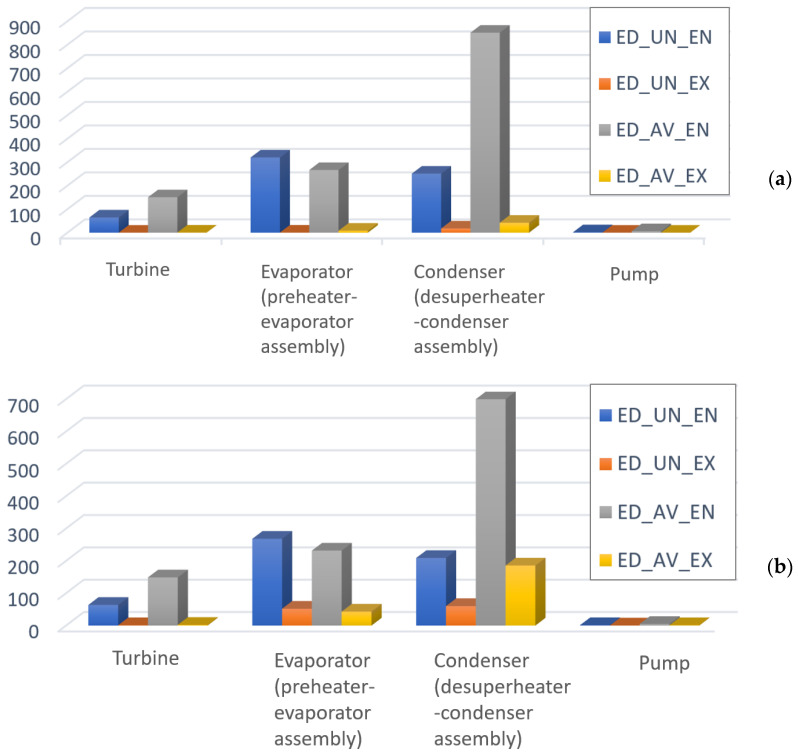
Splitting of the exergy destruction (kW) for the ORC system components: (**a**) ${\dot{Q}}_{sys}=const$, (**b**) ${\dot{W}}_{net}=const$.

Figure 4 presents the results of splitting the exergy destruction into avoidable endogenous and avoidable exogenous parts. These results provide essential information for the designer to better understand the inefficiencies within the system components and their interdependencies. The largest endogenous avoidable exergy destruction is within the condenser, followed by the evaporator and turbine. The exergy destruction of the condenser accounted for more than 65% of the total endogenous avoidable exergy destruction. It can be noted that for all components, the unavoidable exogenous exergy destruction is smaller than the unavoidable endogenous parts. This is the confirmation of very weak interactions among components of the evaluated ORC system.

**Figure 5:**
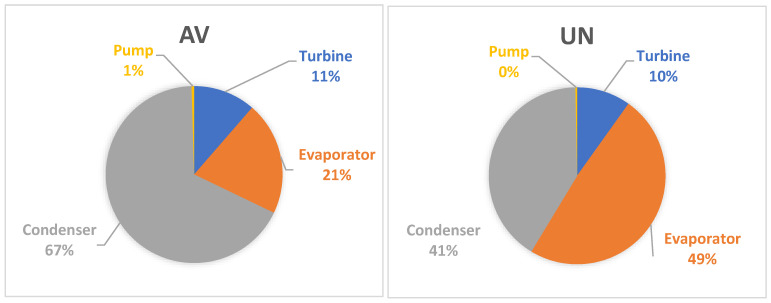

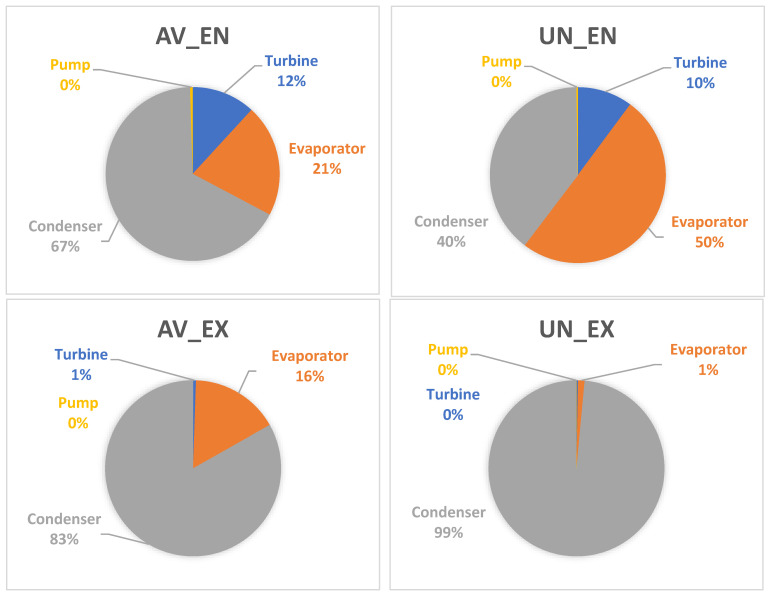
Contribution of each component on cycle overall exergy destruction rate obtained from advanced exergy analysis for ${\dot{Q}}_{sys}=const$.

According to Figure 5, the most important components from the thermodynamic point of view are the condenser and evaporator; the designer should focus first on these components in optimizing the system performance.

The obtained data can be compared to the results from [23], where the advanced exergetic analysis has been applied for the ORC with nine organic working fluids. The results indicate that the most important components are the evaporator and condenser. Unfortunately, the conditions of the analysis are not mentioned.

### 6.2. Advanced Exergoeconomic Analysis

According to Table 4, the endogenous investment cost rate is higher than the exogenous rate. More than 90% of the investment cost of the turbine, condenser, evaporator, and pump is endogenous, which means that only individual operating conditions of the components are affected.

The difference between endogenous and exogenous absolute values of investment cost rates is important. This shows that the investment cost of the component under consideration is mainly affected by the internal thermodynamic inefficiencies and far less by the structure of the system and the operation of the rest of the components.

For all components, the avoidable cost of exergy destruction is higher than the unavoidable cost; this means significant potential for cost reduction for these components. The most relevant component is the condenser, which has 77% of the avoidable cost of exergy destruction. The cost of exergy destruction is mainly endogenous for all the components.

**Figure 6:**
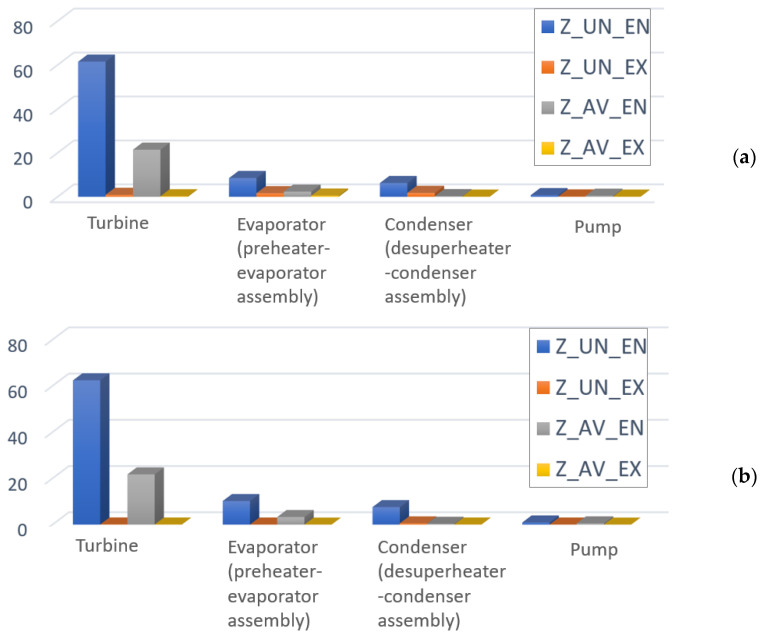
Detailed splitting of the investment cost Z (USD/h): (**a**) ${\dot{Q}}_{sys}=const$, (**b**) ${\dot{W}}_{net}=const$.

**Figure 7:**
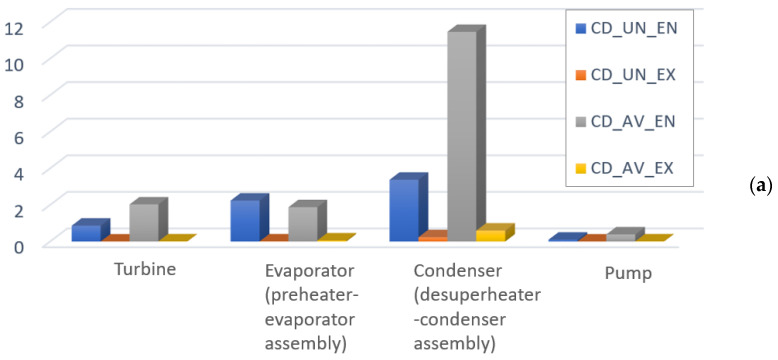

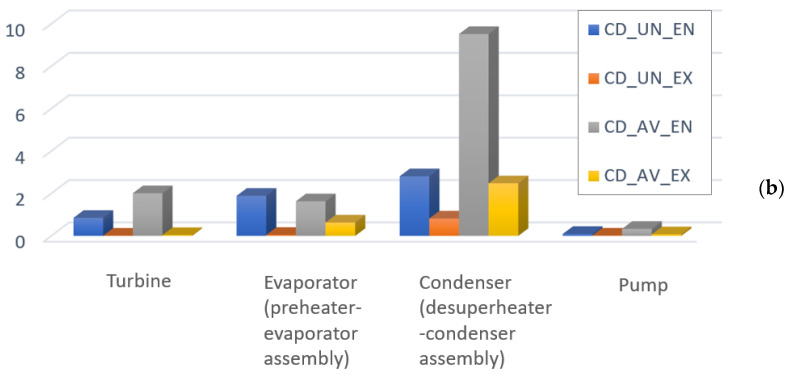
Detailed splitting of the cost of exergy destruction ${\dot{C}}_{D,k}^{}$, (USD/h): (**a**) ${\dot{Q}}_{sys}=const$, (**b**) ${\dot{W}}_{net}=const$.

The results of splitting the investment cost and cost of exergy destruction into endogenous/exogenous parts in combination with the splitting into endogenous/exogenous parts are provided in Figure 6 and Figure 7, and Table 2 reports the main variables. It can be seen that the avoidable endogenous investment cost plays a more significant role in the sum (${\dot{Z}}_{k}^{AV,EN}+{\dot{C}}_{D,k}^{AV,EN}$) for the turbine. The values of $f_{k}^{AV,EN}$ demonstrate that 91.5% of the total avoidable endogenous cost associated with the turbine is investment cost. For the condenser, the value $f_{k}^{AV,EN}$ shows that 3% of the total avoidable endogenous cost is investment cost. Therefore, more than 95% of the performance improvement should be prioritized instead of reducing the investment cost of the component. In the case of the evaporator and pump, it can be seen that both performance and investment costs should be considered in the optimization.

### 6.3. Optimization

**Figure 8:**
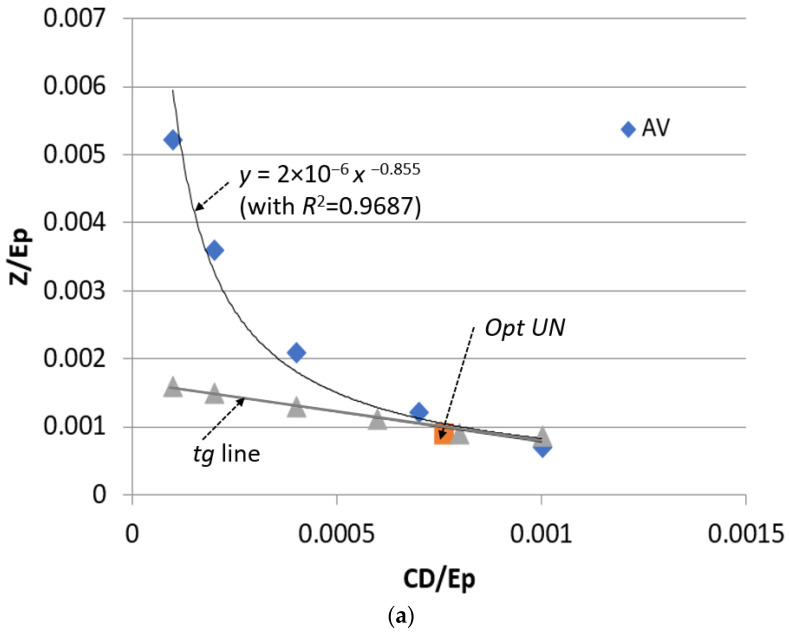

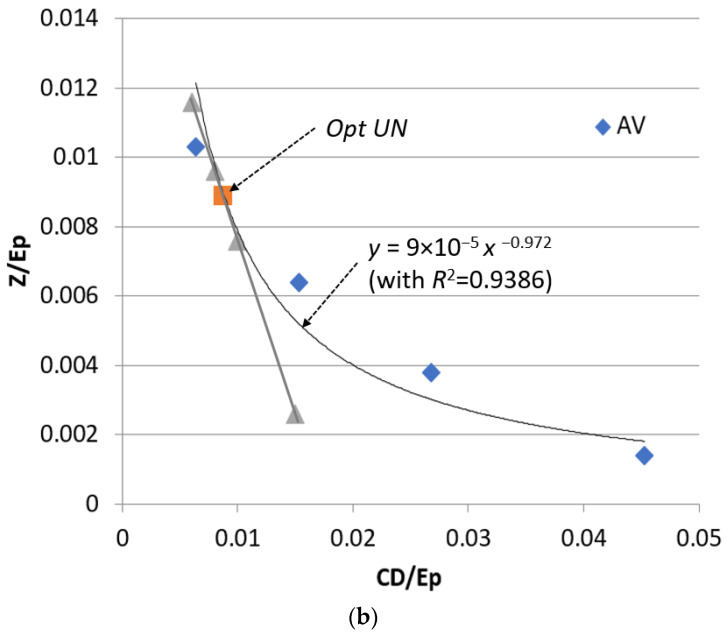
Advanced exergoeconomic optimization for evaporator (**a**) and condenser (**b**).

A graphical optimization procedure is applied for the most critical components: the evaporator and condenser. The curves $\frac{{\dot{Z}}_{k}}{{\dot{E}}_{P,k}}$ vs. $\frac{{\dot{C}}_{D,\;k}}{{\dot{E}}_{P,k}}$ are given for the evaporator and condenser (Figure 8), and limited to avoidable values only, i.e., correspond to blue color symbols in Figure 3 (with approximation equations and R^2^). In addition, the tangent lines and optimal points are provided. The optimal operation conditions show that the evaporator can be improved by increasing the minimum temperature difference from 20.5 K to 21.4 K, which is technically possible. The condenser requires a significant improvement; the minimum temperature difference in this component should be decreased from 37.04 K to 10.0 K.

After applying the obtained optimal conditions for the evaporator and condenser, the overall exergetic efficiency of the ORC system (${\dot{Q}}_{sys}=const$) is increased by 2.5% from *ε_tot_* = 27.1% (Base Case) to *ε_tot_* = 27.7% (Optimal Case), and the specific cost of the generated electricity is decreased by 0.3% from *c*_*P*,*tot*_ = 18.17 USD/GJ (Base Case) to *c*_*P*,*tot*_ = 18.09 USD/GJ (Optimal Case).

## 7. Conclusions

This paper discusses the application of the advanced exergy and exergoeconomic analysis to ORC waste heat recovery. For the first time, the advanced exergy analysis has been applied to the same system but under different operation scenarios: constant heat rate supplied to the ORC or constant power generated by the ORC.

Key results can be summarized as:

- In all the system components, the avoidable exergy destruction is higher than the unavoidable part, which indicates the real perspectives for improving the system thermodynamically.
- The interactions among components of the ORC are not strong. Despite slightly different values obtained for the different conditions (constant heat rate supplied to the ORC or constant power generated by the ORC), the conclusions are the same.
- More than 90% of the investment cost of all the system components is endogenous.
- The components with the highest potential for efficiency and cost improvement are the heat exchangers: evaporator and condenser.
- The graphical method of advanced exergy-based optimization can provide the operation conditions that increase the exergetic efficiency and decrease the cost of the product.

## Figures and Tables

**Table 1:** Definitions used for exergetic and exergoeconomic analysis for the ORC system (TCI values are adapted from [16,20,21]).

Component	Exergy of Fuel, ${\dot{E}}_{F,k}$	Exergy of Product, ${\dot{E}}_{P,k}$	Cost Balance Equations, Auxilary Equations, and *TCI_k_*
Pump	${\dot{W}}_{p}$	${\dot{E}}_{2}-{\dot{E}}_{1}$	${\dot{E}}_{1}c_{1}+{\dot{C}}_{{\dot{W}}_{p}}+{\dot{Z}}_{p}={\dot{E}}_{2}c_{2}$ $c_{{\dot{W}}_{p}}=c_{{\dot{W}}_{tur}}$ ${TCI}_{p}=422{\dot{W}}_{p}^{0.71}\left[1.41+1.41\left(\frac{1-0.8}{1-\eta_{p}}\right)\right]$
Turbine	${\dot{E}}_{4}-{\dot{E}}_{5}$	${\dot{W}}_{tur}$	${\dot{E}}_{4}c_{4}+{\dot{Z}}_{tur}={\dot{E}}_{5}c_{5}+{\dot{C}}_{Wtur}$ $c_{4}=c_{5}$ ${TCI}_{tur}=6000\;{\dot{W}}_{tur}^{0.7}$
Preheater	${\dot{E}}_{8}-{\dot{E}}_{9}$	${\dot{E}}_{3}-{\dot{E}}_{2}$	${\dot{E}}_{2}c_{2}+{\dot{E}}_{8}c_{8}+{\dot{Z}}_{pre}={\dot{E}}_{3}c_{3}+{\dot{E}}_{9}c_{9}$ $c_{8}=c_{9}$ ${TCI}_{pre}=10000+324{\;A}^{0.91}$
Evaporator	${\dot{E}}_{7}-{\dot{E}}_{8}$	${\dot{E}}_{4}-{\dot{E}}_{3}$	${\dot{E}}_{3}c_{3}+{\dot{E}}_{7}c_{7}+{\dot{Z}}_{eva}={\dot{E}}_{4}c_{4}+{\dot{E}}_{8}c_{8}$ $c_{7}=c_{8}$ ${TCI}_{eva}=10000+324{\;A}^{0.91}$
Desuperheater	${\dot{E}}_{5}-{\dot{E}}_{6}$	${\dot{E}}_{13}-{\dot{E}}_{12}$	${\dot{E}}_{5}c_{5}+{\dot{E}}_{12}c_{12}+{\dot{Z}}_{desup}={\dot{E}}_{6}c_{6}+{\dot{E}}_{13}c_{13}$ $c_{5}=c_{6}$ ${TCI}_{desup}=10000+324{\;A}^{0.91}$
Condenser	${\dot{E}}_{6}-{\dot{E}}_{1}$	${\dot{E}}_{12}-{\dot{E}}_{11}$	${\dot{E}}_{6}c_{6}+{\dot{E}}_{11}c_{11}+{\dot{Z}}_{con}={\dot{E}}_{1}c_{1}+{\dot{E}}_{12}c_{12}$ $c_{6}=c_{1}\;c_{11}=0\;$ ${TCI}_{con}=10000+324{\;A}^{0.91}$
IHE	${\dot{E}}_{g,in}-{\dot{E}}_{g,out}$	${\dot{E}}_{7}-{\dot{E}}_{10}$	${\dot{C}}_{g,in}+{\dot{E}}_{10}c_{10}+{\dot{Z}}_{IHE}={\dot{C}}_{g,out}+{\dot{E}}_{7}c_{7}$ $c_{g,in}=c_{g,out}$ ${TCI}_{IHE}=10000+324{\;A}^{0.91}$

**Table 2:** Data obtained from the conventional and advanced exergy-based analyses for the ORC system components at ${\dot{Q}}_{sys}=const$.

	${\dot{E}}_{D,k}$ (kW)	$\dot{Z_{k}}/$ ${\dot{Z}}_{k}^{AV,EN}$ (USD/h)	${\dot{C}}_{D,k}/$ ${\dot{C}}_{D,k}^{AV,EN}$ (USD/h)	*f_k_*/ $f_{k}^{AV,EN}$ (%)
Turbine	215.78	84.23/21.75	2.91/2.03	97%/91%
Preheater–Evaporator assembly	595.33	13.48/2.27	4.19/1.88	76%/55%
Desuperheater–Condenser assembly	1162.90	8.49/0.36	15.68/11.46	35%/3%
Pump	7.76	1.47/0.54	0.51/0.39	75%/58%

**Table 3:** Values of parameters assumed for the different operation conditions of the ORC system.

Component	Parameter	Real Conditions	«Best»/«Worse» Conditions	Ideal Conditions
Turbine	*η* (%)	85	95/70	100
Evaporator	∆*T_min_* (K)	20	5/30	0
Condenser	∆*T_min_* (K)	37	5/40	0
Pump	*η* (%)	70	95/65	100

**Table 4:** Advanced exergetic and exergoeconomic analyses.

	Unavoidable and Avoidable Parts						Endogenous and Exogenous Parts ${\dot{Q}}_{sys}=const$$/{\dot{W}}_{net}=const$					
	${\dot{E}}_{D,k}^{UN}$ (kW)	${\dot{E}}_{D,k}^{AV}$ (kW)	${\dot{Z}}_{k}^{UN}$ (USD/h)	${\dot{Z}}_{k}^{AV}$ (USD/h)	${\dot{C}}_{D,k}^{UN}$ (USD/h)	${\dot{C}}_{D,k}^{AV}$ (USD/h)	${\dot{E}}_{D,k}^{EN}$ (kW)	${\dot{E}}_{D,k}^{EX}$ (kW)	${\dot{Z}}_{k}^{EN}$ (USD/h)	${\dot{Z}}_{k}^{EX}$ (USD/h)	${\dot{C}}_{D,k}^{EN}$ (USD/h)	${\dot{C}}_{D,k}^{EX}$ (USD/h)
Turbine	64.81	150.96	62.46	24.70	0.87	2.03	215.45/ 212.76	0.33/ 3.02	84.15/ 82.99	0.08/ 1.15	2.90/ 2.86	0.01/ 0.05
Evapo-rator	320.34	274.98	10.21	3.21	1.93	2.25	586.79/ 500.01	8.53/ 95.32	13.47/ 11.28	0.01/ 2.19	4.12/ 3.52	0.07/ 0.67
Con-denser	269.30	893.59	8.11	0.37	3.62	12.05	1102.6/ 916.92	60.3/ 245.97	7.93/ 6.59	0.56/ 1.90	14.84/ 12.34	0.84/ 3.33
Pump	1.97	6.08	0.92	0.54	0.11	0.40	7.75/ 6.45	0.01/ 1.31	1.46/ 1.22	0.01/ 0.24	0.5/ 0.42	0.01/ 0.09

## Data Availability

The data presented in this study are available on request from the corresponding authors.

## Nomenclature

*A*	heat transfer area (m^2^)
$\dot{C}$	cost rate (USD/h)
*c*	cost per exergy unit (USD/GJ)
$\dot{E}$	exergy rate (kW)
f	exergoecononmic factor (%)
$\dot{m}$	mass flow rate (kg/s)
*p*	pressure (bar)
*T*	temperature (°C or K)
*TIC*	total capital investment (USD)
$\dot{W}$	power (kW)
$\dot{Z}$	capital investment cost rate (USD/h)
*Subscripts*	
1, 2, …	system state points
*con*	condenser
*D*	destruction
*desup*	desuperheater
*eva*	evaporator
*F*	fuel
*k*	*k*th component
*L*	loss
*P*	product
*p*	pump
*pre*	preheater
*recp*	recuperator
*tot*	system
*tur*	turbine
*wf*	working fluid of ORC
*Greek letters*	
*η*	isentropic efficiency of turbine and pump (%)
*ε*	exergy efficiency (%)
*Abbreviations*	
HRVG	heat recovery vapor generator
IHE	intermediate heat exchanger
ORC	Organic Rankine Cycle
WHR	waste heat recovery
